# Is There Evidence for Synergy Among Air Pollutants in Causing Health Effects?

**DOI:** 10.1289/ehp.11654

**Published:** 2008-08-22

**Authors:** Joe L. Mauderly, Jonathan M. Samet

**Affiliations:** 1 National Environmental Respiratory Center, Lovelace Respiratory Research Institute, Albuquerque, New Mexico, USA; 2 Department of Epidemiology, Johns Hopkins Bloomberg School of Public Health, Baltimore, Maryland, USA

**Keywords:** air pollution, combined exposures, mixtures, ozone, synergy

## Abstract

**Background:**

Environmental air pollutants are inhaled as complex mixtures, but the long dominant focus of monitoring and research on individual pollutants has provided modest insight into pollutant interactions that may be important to health. Trends toward managing multiple pollutants to maximize aggregate health gains place increasing value on knowing whether the effects of combinations of pollutants are greater than the sum of the effects of individual pollutants (synergy).

**Objective:**

We reviewed selected published literature to determine whether synergistic effects of combinations of pollutants on health outcomes have actually been demonstrated.

**Methods and results:**

We reviewed 36 laboratory studies of combinations of ozone with other pollutants that were reported in the recent U.S. Environmental Protection Agency Ozone Criteria Document. We examined original reports to determine whether the experimental design tested for synergy and whether synergy was demonstrated. Fourteen studies demonstrated synergism, although synergistic, additive, and antagonistic effects were sometimes observed among different outcomes or at different times after exposure.

**Conclusions:**

Synergisms involving O_3_ have been demonstrated by laboratory studies of humans and animals. We conclude that the plausibility of synergisms among environmental pollutants has been established, although comparisons are limited, and most involved exposure concentrations much higher than typical of environmental pollutants. Epidemiologic research has limited ability to address the issue explicitly.

Air pollution exists as a complex mixture that varies spatially and temporally in its characteristics. In the United States and other countries, major air pollutants are regulated individually, although when present in a mixture, their toxicity could differ from that found in investigations directed at individual pollutants. As recommended by the National Research Council (NRC 2004a), there is increasing interest in managing environmental air quality using multipollutant strategies aimed at reducing the aggregate health burden of air pollution. Therefore, from both the public health and regulatory perspectives, the potential for synergy among mixture components is a particular concern. A fair and increasingly frequent question is whether there are any documented examples of synergies among air pollutants. That question was the focus of our review and this commentary.

## Definitions and strategies

Synergy is strictly defined as occurring if the effect of the combined exposure is greater than the sum of the effects of the two or more individual components of the mixture (see definitions in Appendix 1). The term is used loosely and sometimes applied to any effect of a combination of pollutants that is greater than the effect of one of the components alone. This circumstance is not an instance of synergy as defined within the public health community unless the effects are greater than additive. The term also extends to an effect caused by a combination of pollutants but not caused by exposure to the individual pollutants, absent exposure to the others. This applied definition makes clear the need to have evidence on both the individual and the combined effects of any combination of pollutants to evaluate the presence of synergism.

The term “interaction” also requires a careful definition. As defined in statistical modeling, interaction refers to the interdependence of the effects of two or more variables. Product terms of the potentially interacting variables are inserted into analytical models to test for the presence of interaction, which may be synergistic or antagonistic. The statistical tests for interaction have low statistical power, and consequently the joint effects of several factors may be only imprecisely characterized, unless there is a strong interaction or abundant data. Often, the term “interaction” is used loosely to refer to synergism or antagonism, although more accurately, interactions can be characterized as synergistic or antagonistic, depending on the direction of the combined effect. Epidemiologists also use the term “effect modification” to refer to interdependence of the effects of two or more variables (Last 2000).

Synergism between two environmental pollutants might occur through a variety of mechanisms. First, the two pollutants might act at the same or different steps in the same mechanistic pathway; second, the presence of one might influence ability to mitigate the action of the other; and third, the presence of one might influence the dose of the other. There are the possibilities that the existence of synergism could be dose dependent, that the same combined exposure might be synergistic for one effect and not for others, or that the same effect may be synergistic in some tissues and not in others, as illustrated by examples presented later.

Laboratory and epidemiologic studies have demonstrated effects of combined or sequential exposures that were greater than the effects of either exposure given singly, but the combined effects were often simply additive. Other such studies have demonstrated combined effects that were less than additive. A strict quantitative test of synergy requires measurement of the effects of each component and of the combined components administered under identical conditions. For environmental exposures studied with epidemiologic methods, the corresponding conditions would include sufficiently comparable populations, measurements of effect, coexposures to other pollutants, and other modifying factors. Such identical conditions are impossible to achieve in the strictest sense, but may be approximated to varying degrees in epidemiologic studies through careful design and analysis.

In assessing synergism among pollutants using epidemiologic approaches, the minimum information needed is estimation of exposure or dose for the two or more pollutants under investigation. Most often, synergism is assessed using multivariable models that include terms representing the effects of the individual pollutants and one or more interaction terms that represent potential joint effects (Greenland 1983; Rothman et al. 2008). A model for two pollutants would be as follows:


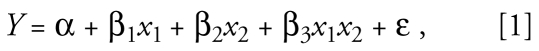


where *Y* is the outcome, *x*_1_ and *x*_2_ are two specific pollutants, β_1_ and β_2_ estimate the pollutant-specific effects of *x*_1_ and *x*_2_, and β_3_ estimates the joint effect of *x*_1_ and *x*_2_, and ɛ is the error term. In the presence of interaction (synergism is positive interaction), β_3_ is not zero, the value indicating no interaction, and the effect of *x*_1_ is *x*_1_(β_1_ + β_3_*x*_1_*x*_2_) and that of *x*_2_ is *x*_2_(β_2_ + β_3_*x*_1_*x*_2_). In the example of smoking, radon exposure, and lung cancer, *Y* might represent risk for lung cancer death, *x*_1_ the cumulative amount smoked, and *x*_2_ the cumulative exposure to radon. For three pollutants, there are three two-way interaction terms (for *x*_1_*x*_2_, *x*_1_*x*_3_, and *x*_2_*x*_3_) as well as a three-way interaction term for *x*_1_*x*_2_*x*_3_. As the number of potentially interacting pollutants increases, the number of possible interaction terms increases progressively. The coefficients are estimated by the algorithm used to fit the model to the data—for example, least squares or maximum likelihood.

Even for two exposures, statistical power is limited for detecting interactions, and the value of β_3_ is estimated with limited precision, unless the data are substantial or there is particularly strong synergism (Greenland 1983; Rothman et al. 2008). If interactions among three or more pollutants are of interest, power is likely to be extremely limited, given the number of terms in the model. Interpretation of models directed at interaction also needs to consider error in the measurement of the pollutants and the consequences of errors that are differential among the pollutants. A further consideration is the scale on which the interaction is assumed to take place: additive or multiplicative. Typically, additive models are used for continuous outcome measures, such as lung function level, whereas multiplicative models are used for risk for events, such as probability of dying. Interpretation of interaction terms is scale dependent, although for public health purposes, epidemiologists are in consensus that positive departure from additivity constitutes synergism (Rothman et al. 2008). Although the scale of interaction should be based in biological understanding, there is not a specific correspondence between biological interaction and statistical interaction in the modeling of epidemiologic data.

In the laboratory, evaluating synergism between pollutants A and B, for example, would require four exposure scenarios: *a*) control, *b*) A alone, *c*) B alone, and *d*) A + B (each at the same concentration as when given singly). Either the same or different groups of subjects might receive each treatment, depending on the nature of the treatment, persistence of effects, and measurement methods.

## Background knowledge

The Health Effects Institute convened a working group during 1990–1992 to review the subject (Samet and Speizer 1993a), and information in the resulting papers described challenges and strategies for evaluating complex mixtures by epidemiologic (Dockery 1993; Samet and Speizer 1993b; Weiss 1993) and laboratory studies (Mauderly 1993; McDonnell 1993). The NRC and other organizations have convened numerous committees on the related topic of complex mixtures. Beyond subsequent reports of several laboratory studies examining interactions among pollutants at high doses, it is not clear that the understanding of synergistic interactions among environmental pollutants has advanced substantially beyond the 1993 reports.

Synergisms among occupational inhalation exposures are known; perhaps the best documented example is the synergy between radon progeny and cigarette smoking in producing lung cancer in underground miners (NRC 1988, 1999). In this example, the presence of synergism was shown by a pooled analysis of data that contained information from cohorts of underground miners on both exposure to radon progeny and smoking. Statistical models were used to estimate the degree of synergism, which could be determined with reasonable precision because substantial data were available. Well-studied examples of potential synergism between smoking and other disease risk factors include asbestos and lung cancer [Agency for Toxic Substances and Disease Registry (ATSDR) 2006; Selikof et al. 1968] and oral contraceptives and pulmonary thromboembolism (Lidegaard 1999; Petitti et al 1996).

A review by the ATSDR of the toxicologic and epidemiologic literature on effects of mixtures (ATSDR 2004) found some, but scant, evidence for synergisms from exposures to multiple chemical agents. In addition to examples in which each component caused measurable effects, examples were cited in which combined exposures caused measurable effects that were not observed when the components were administered individually at the same doses. Among the combined exposures reviewed by ATSDR, the effects were more commonly less than additive, rather than greater than additive.

The air pollution literature yields similarly mixed results. For example, Chen et al. (1991) observed greater than additive effects on the pulmonary function of guinea pigs from exposure to sulfuric acid (H_2_SO_4_), acid-coated zinc oxide particles, and ozone. Kleinman et al. (2000) observed less than additive effects on the proliferation of the respiratory epithelium of rats exposed to O_3_, carbon particles, and ammonium bisulfate given singly and combined. Anderson and Avol (1992) did not find significant synergy between the effects of carbon particles and H_2_SO_4_ on the respiratory function of experimentally exposed humans; however, the combination elicited effects in some subjects having no response to either carbon or acid alone, suggesting possible synergy. Jakab and Hemenway (1994) found that the effects of inhaled O_3_ and carbon black were synergistic in causing lung inflammation and suppressing phagocytosis by alveolar macrophages recovered from rats in bronchoalveolar lavage fluid (BALF). The effects of carbon black and O_3_ were synergistic only when inhaled together, not when administered sequentially. Creutzenberg et al. (1995) found the combined effect of inhaled O_3_ and instilled carbon black on the uptake of particles by cells collected from exposed rats in BALF to be less than additive, but the effect on the migration of cells in response to a chemoattractant was synergistic. The latter two studies demonstrated that effects of combined and sequential exposures might differ and that the same exposure may be synergistic for some, but not all, outcomes.

There have also been studies of interactions between air pollutants and other agents, and these, too, have produced mixed results. For example, Spannhake et al. (2002) found a greater than additive release of the proinflammatory cytokine interleukin-8 from the BEAS-2B human airway epithelial cell line treated with rhinovirus and exposed to either O_3_ or nitrogen dioxide. Harrod et al. (2003) found less than additive effects on bronchoalveolar lavage cell counts, proinflammatory cytokines, and Clara cell secretory protein levels in mice exposed to respiratory syncytial virus and diesel emissions. One could also consider many of the studies of the effects of pollutant exposure on airway reactivity as tests of synergy, if they include exposures to the pollutant and the airway agonist alone as well as in combination. For example, several of the 29 studies listed in Table AX6-11 (Airway Responsiveness Following Ozone Exposure) of the most recent U.S. Environmental Protection Agency (EPA) Criteria Document for O_3_ (U.S. EPA 2006) fall into this category, and by strict definition, demonstrate synergy between O_3_ and the airway agonist.

## Review of Combined Exposure Studies Cited in 2006 U.S. EPA Ozone Criteria Document

The above studies indicate that synergies involving environmental air pollutants have been demonstrated in the laboratory and that O_3_ has been a component of synergistic combinations of pollutants. We turned to the 2006 U.S. EPA Criteria Document for O_3_ (U.S. EPA 2006) to examine in detail a larger set of studies of combined exposures involving O_3_. This subset of the air pollution literature is a potentially informative body of evidence because it has been assembled in a policy-directed document. O_3_ is a common pollutant that has been studied extensively in population and laboratory settings, and the three tables encompass a sizable number of studies addressing the combined effects of O_3_ and other pollutants. The original publications were examined to determine whether the study designs provided tests of synergy and, if so, whether a positive departure from additivity was demonstrated. The 14 studies we found to demonstrate synergies are listed in Table 1.

We did not attempt a more comprehensive review of the air pollution literature, although such a review would be useful. People are certainly exposed to a wide spectrum of additional pollutant combinations, and the effects of some internally complex pollutant classes such as particulate matter (PM) may involve synergisms among components. We found that our limited review was sufficient to solidly confirm the answer to the question of whether synergies among pollutants found in the environment had been demonstrated.

### Laboratory studies of humans exposed to combinations including O_3_

We examined 19 papers cited in Table AX6-14 (Ozone Mixed with Other Pollutants) of the 2006 U.S. EPA Criteria Document. We found that the designs of 13 of 19 studies demonstrating significant effects were adequate to test for synergy. Only the following two studies (Table 1) demonstrated effects of combined exposures to O_3_ and another pollutant that were greater than the sum of the effects of the individual pollutants. The other 11 studies testing for synergy did not demonstrate effects that were greater than additive.

Horvath et al. (1986) exposed young, nonsmoking women for 2 hr during intermittent exercise to 485 ppb O_3_, to 2 ppb peroxyacetyl nitrate (PAN), and to the combination of O_3_ and PAN. Measurements included heart rate, breathing pattern, lung volumes, forced exhalation, and O_2_–CO_2_ exchange. PAN alone caused some symptoms but either no or very small, insignificant effects among measured variables. O_3_ caused more reports of symptoms and significant effects on several measured variables. The combined exposure was additive with respect to total reported symptoms but greater than additive for most affected variables. For example, forced vital capacity near the end of exposure was reduced 2% by PAN, 23% by O_3_, and 31% by the combination, indicating a combined effect 24% greater than additive.

Drechsler-Parks (1995) exposed healthy older adults (five men and one woman, 56–85 years of age, completed the protocol) for 2 hr during intermittent exercise to 450 ppb O_3_, 600 ppb NO_2_, or to O_3_ and NO_2_ combined. Measurements included respiration, heart rate, cardiac output, and stroke volume. Cardiac output was increased 1% by O_3_, reduced 5% by NO_2_, and reduced 14% by the combination. Stroke volume was reduced 2% by O_3_, reduced 7% by NO_2_, and reduced 12% by the combination. These results suggest modest synergy.

### Laboratory studies of animals exposed to combinations including O_3_

We examined 17 papers listed as reporting synergistic effects in Tables AX5-17 (Interactions of Ozone with Tobacco Smoke) and AX5-18 (Interactions of Ozone with Particles) of the U.S. EPA Criteria Document (U.S. EPA 2006). We found that only 14 of 17 studies were designed to test for synergism, and that only the 12 listed in Table 1 actually demonstrated synergism by reporting effects of combined exposures that were greater than the sum of effects of the individual exposures. This discrepancy between the authors’ conclusions and our review reflects the common tendency to use the term “synergism” loosely. The 12 studies demonstrating synergism for at least one measured outcome are described briefly here.

Two studies examined interactions between O_3_ and acid aerosols. Kimmel et al. (1997) exposed Sprague-Dawley rats 4 hr/ day for 2 days to O_3_ at 600 ppb, to fine (300 nm) or ultrafine (60 nm) H_2_SO_4_ aerosol at 500 μg/m^3^, or to the combinations of O_3_ and each of the acid aerosols and performed morphometric measurements of cell proliferation and damage to alveolar septa. The volume percentage of markedly damaged alveolar tissue was only slightly increased by either acid exposure but markedly increased by O_3_. The combined effects were greater than additive. A similar pattern was observed for cell proliferation, but the degree of synergy was less pronounced.

Sindhu et al. (1998) exposed Fischer-344 (F344) rats 4 hr/day, 3 days/week for 40 weeks to O_3_ at 150 ppb, nitric acid (HNO_3_) at 50 μg/m^3^, or the combination, and measured the lung content of polyamines. O_3_, but not HNO_3_, increased lung putrescine, and the combined effect was 2-fold greater than the O_3_ effect. Both exposures also increased lung spermidine and spermine contents, but the combined effects were less than additive.

Two studies examined interactions between O_3_ and aerosols of resuspended PM collected in Ottawa, Ontario, Canada. Vincent et al. (1997) exposed F344 rats for 4 hr to either 800 ppb O_3_; 5,000 or 48,000 μg/m^3^ of resuspended urban PM from Ottawa (EHC-93); or to the combination of O_3_ and each concentration of particles. They evaluated cell proliferation in the lung parenchyma and bronchiolar region by cell labeling. Only O_3_ increased cell labeling when given alone. The combined effects were synergistic for labeling in the bronchioles but approximately additive for labeling in whole-lung parenchyma.

Adamson et al. (1999) exposed F344 rats for 4 hr to either 800 ppb O_3_, 57,000 μg/m^3^ PM, or to the combination and evaluated cell proliferation in the whole lung and alveolar duct region by cell labeling. As in the former study, only O_3_ increased cell labeling when given alone, and the combined effects were approximately additive for whole-lung labeling; however, the effect was clearly synergistic (approximately 4-fold) for labeling in the alveolar duct region.

Two studies examined interactions between O_3_ and cigarette smoke, a complex mixture encountered in indoor and outdoor environments. Wu et al. (1997) exposed guinea pigs to a few breaths (two puffs) of diluted cigarette smoke followed by exposure to 1,500 ppb O_3_ for 1 hr and then measured dynamic lung compliance and resistance. The effects of smoke and O_3_ alone were compared with the effects of the sequential combination. Compliance was reduced by both exposures, and the combined effect was slightly greater than additive. Resistance was increased only by smoke when exposures were given singly, but the increase due to combined exposure was approximately 10-fold greater than the effects of smoke alone.

Yu et al. (2002) exposed B6C3F1 mice 6 hr/ day for 3 days to aged, diluted side-stream cigarette smoke at 30,000 μg PM/m^3^ and then to 500 ppb O_3_ for 24 hr, or to the two pollutants alone, followed by measurement of centriacinar cell proliferation and markers of inflammation in BALF. Both exposures increased BALF neutrophils, and the combined effect was decidedly greater than additive. Neither exposure increased BALF lymphocytes, but the combined exposure elicited a nearly 2-fold increase over control levels. The effects on BALF tumor necrosis factor alpha were slightly synergistic, and the effects on BALF interleukin-8 and cell proliferation were additive.

Five studies examined interactions between O_3_ and endotoxin. Although endotoxin is not a commonly measured air pollutant, it is ubiquitous in the environment and a frequent component of environmental PM. Johnston et al. (2002) exposed C57BL6J mice for 24 hr to O_3_ at 1,000 ppb, to aerosolized endotoxin at an estimated lung dose of 37.5 EU, or to O_3_ followed by endotoxin, and measured responses in BALF at 4 and 24 hr. At 4 hr, there were slightly synergistic increases in interleukin-1 β and interleukin-6, an additive effect on interleukin-1Ra, and less than additive effects on interleukin-1α and macrophage inhibitory factor. However, the effects on all of the markers were slightly to markedly synergistic at 3 days.

One group reported three different studies using identical sequences of exposure to O_3_ followed by intranasal instillation of endotoxin, including groups receiving the individual treatments. Fanucchi et al. (1998) exposed F344 rats 8 hr/day for 3 days to 500 ppb O_3_ followed by two daily intranasal instillations of endotoxin and evaluated nasal tissues by immunohistochemistry and RNA analysis at 3 hr or 6 days. Endotoxin alone had little effect on the volume density of AB/PAS-staining mucosubstances, but O_3_ increased staining. The combined effect was somewhat synergistic at 6 hr and strikingly synergistic (> 6-fold) at 3 days after exposure. The effect on expression of the *rMuc-5AC* gene was approximately additive at 6 hr, but synergistic (3-fold) at 3 days. Using the same exposure protocol in two subsequent studies of F344 rats, Wagner et al. (2001a, 2001b) demonstrated the repeatability of the synergistic effect on nasal mucosubstances.

In another study by the same group, Wagner et al. (2003) exposed F344 rats 8 hr/ day for 2 days to 1,000 ppb O_3 ,_ preceded each day by intranasal instillation of 2 or 20 μg endotoxin, and included groups receiving the individual treatments. Three days later, they examined effects in the lung by analysis of BALF and changes in airway epithelium. The exposures produced synergistic increases in BALF neutrophils, mucin glycoprotein, and elastase, and also in intraepithelial mucosubstances and epithelial cell density in distal airways. Increases in BALF lymphocytes and macrophages were approximately additive, and the effect on epithelial cell density in proximal airways was less than additive.

One study examined interactions between O_3_ and ovalbumin instilled intranasally. Wagner et al. (2002) exposed Brown Norway rats to 500 ppb O_3_ for 1 or 3 days and instilled 50 μL 1% ovalbumin after each O_3_ exposure. At 24 hr after the last treatment, they examined inflammatory and epithelial cell populations, the volume densities of intra-epithelial mucosubstances, and cell proliferation rates in nasal epithelium. The effect of O_3_ and antigen on epithelial mucosubstances was synergistic in the maxilloturbinates but less than additive in the septum. The effect on eosinophil influx was also synergistic in the maxilloturbinates but less than additive in the septum. The combined effects were less than additive for neutrophil influx, cell labeling, and epithelial cell density.

## Summary, Discussion, and Conclusions

Our examination of studies cited in the 2006 O_3_ Criteria Document (U.S. EPA 2006) confirmed that synergisms between O_3_ and other pollutants have been demonstrated in laboratory studies involving humans and animals. Fourteen studies among the 13 human and 14 animal studies testing for synergy demonstrated greater than additive effects for one or more outcomes. The co-pollutants in these studies included urban PM, cigarette smoke, H_2_SO_4_, HNO_3_, NO_2_, PAN, endotoxin, and antigen. These co-pollutants are all plausible classes of environmental air contaminants (accepting the neoantigen ovalbumin as a model for environmental proteinaceous antigens). The additional studies not contained in the 2006 O_3_ Criteria Document, but cited above as examples of synergy involving O_3_, included carbon black, virus, and airway agonists. This limited review, therefore, identified diverse examples of synergies between O_3_ and other air pollutants, as well as synergies involving combinations of H_2_SO_4_ with carbon particles, and diesel emissions with virus. Of course, environmental exposures involve much more complex mixtures than those typically used in the laboratory. The studies we reviewed involved only a very few of the myriad pollutants encountered in the environment in different combinations. Only the studies including tobacco smoke or diesel emissions approached a realistic level of complexity, and only Kleinman et al. (2000) used a factorial design to examine the single and combined effects of more than two treatments (they included three).

Differences in dose and dose pattern are key caveats in extrapolating these laboratory findings to environmental exposures. Nearly all of the laboratory studies involved high concentrations that are not reflective of typical environmental exposures. The studies of humans involved 2-hr exposures to 450–485 ppb O_3_ and concentrations of PAN and NO_2_ much higher than ambient levels in the United States. The O_3_ exposures of animals ranged from 150 to 1,500 ppb, and only the study by Sindhu et al. (1998) included exposures longer than 3 days. In that study, the exposure of rats 4 hr/day, 3 days/week for 40 weeks to 150 ppb O_3_ ± 50 μg/m^3^ HNO_3_ used concentrations of O_3_ only twice the current 1-hr U.S. National Ambient Air Quality Standard (U.S. EPA 2008), but a concentration of HNO_3_ many-fold higher than is typical of the environment. The publications provide no indication as to whether any of these research groups have explored the dose–response relationships of their published findings down to environmental exposure levels. Unfortunately, the limitation posed by the range of exposure concentrations is a widespread issue in interpreting toxicology studies of air pollution and not only studies of combined exposures.

Existing information yields little indication of whether synergies observed in animals might also occur in humans, in animals of other species, in animals of other strains of the same species, or in subjects of different ages. Interspecies, interstrain, and age-related differences exist in the uptake and metabolism of inhaled materials and in the susceptibility and sensitivity to adverse effects. The fact that more animal than human studies in our review demonstrated synergies cannot be interpreted to mean that synergies are less likely to occur in humans. Our limited survey revealed no studies in which different species, strains, or ages were exposed to the same combinations of pollutants. Like the dose issue, the lack of systematic comparisons among research models is a prevalent limitation throughout the air pollution literature.

The current evidence for synergism is restricted primarily to subclinical responses. The outcomes for which synergism was demonstrated in the studies described above encompassed a diverse spectrum of responses, including indicators of activated gene expression (e.g., *Muc5AC*), chemoprotective responses (e.g., lung polyamines), proinflammatory cytokines (e.g., interleukins), frank inflammation (e.g., BALF neutrophils), alterations of cellular populations and functions (e.g., epithelial proliferation, increased mucosubstances), and alterations of organ-level function (e.g., cardiac output, forced vital capacity). Although these effects reflect perturbations of response pathways that can contribute to health outcomes observed in population studies, only the last (lung function) has been commonly measured in the population studies undergirding health-based air quality regulations. It is possible that some synergisms occurring at intermediate steps in pathogenic pathways may not be manifest in outcomes at the clinical level. For many examples of synergism we identified, it is uncertain whether the synergism would be manifest at clinical and public health scales. Effects at subclinical levels have provided supportive evidence for associations between air pollutants and health, but air quality regulations have been based generally on mortality and morbidity outcomes that are a consequence of the integrated actions of myriad molecular and cellular biological responses. The interpretation of the implications of synergistic effects on subclinical homeostatic and pathologic responses raises the possibly insoluble dilemma of defining at what level measured responses constitute adverse effects (American Thoracic Society 2000). The present information offers only indirect evidence for synergies among pollutants in morbidity or mortality caused or exacerbated by air pollutants.

Ideally, hypotheses related to synergism would be tested under real-world conditions in epidemiologic studies. However, several barriers weaken the epidemiologic approach to investigating synergism. First, the exposures to the multiple pollutants of concern need to be estimated; this may prove difficult because of the complexity of estimating exposures to pollutants as they vary spatially and temporally. Inaccuracies in exposure assessment will tend to decrease the sensitivity of a study for estimating the degree of synergy. Second, unless a high degree of synergism is anticipated, study populations of substantial size are needed to characterize the combined effects of multiple agents because of the limited statistical power of the analytic methods used to assess synergism. It is impractical to test for synergisms in the population as precisely as the laboratory permits. The composition of the environmental air pollution mixture varies in location and time, but the spatial–temporal variation generally does not provide the degree of exposure contrasts needed to compare effects of multiple pollutants alone and in combination. The effects of single pollutants are generally inferred from such variations, but characterization of quantitative relationships is far more imprecise for the effects of combinations of pollutants than for the effects of single pollutants. The precision of exposure estimates also poses a limitation. Accurate measures of personal exposures have only been linked to individual outcomes in panel studies of limited scope, and even then, no study has measured the full range of pollutant species to which the subjects were exposed. The weight of air pollution epidemiology necessarily rests largely on estimates of exposure derived from monitoring data mandated by air quality regulations; thus, substantial data are available for only a few pollutants and pollutant classes.

In this review we identified a need for more rigorous reporting of findings of studies that consider synergism. The term is widely and loosely used. Claims of synergism should be accompanied by the needed quantitative assessment of the evidence and preferably by an assessment of the precision with which synergism has been demonstrated. Our determination of whether synergism was demonstrated in the studies we examined was semiquantitative and often based on graphs rather than numeric data. Reports of statistical significance were limited to differences between effects of combined and sham exposures or between combined and single exposures; the statistical significance with which combined exposures had greater than additive effects was not reported.

The results of this review indicate that synergies among environmental air pollutants are plausible and that experimental approaches may document their existence. It is both plausible and widely accepted that few, if any, effects of air pollution are attributable to single pollutants exclusively, although both epidemiologic and laboratory findings indicate that single pollutants or pollutant classes can dominate certain effects. Nonetheless, research strategies have been driven largely by single-pollutant, single-source regulatory frameworks, and thus have focused on detecting and confirming the causality of single pollutants or specific complex source emissions treated as a single exposure material. Very limited emphasis has been given to apportioning effects among the full spectrum of pollutants or evaluating pollutant interactions. A shift of the emphasis of air pollution health research toward a more comprehensive, forward-thinking, multipollutant perspective would be timely in view of the increasing trend toward multipollutant regulatory strategies. Despite the limitations of laboratory and epidemiologic research tools, both approaches could plausibly be directed more toward a better understanding of the roles of a much broader spectrum of air contaminants, and thus their sources, to the health impacts associated statistically with indices of air pollution. Although it is clearly impossible to study large numbers of pollutants using a full factorial study design, there are multiple strategies for disentangling the contributions of multiple pollutants in the laboratory (Mauderly 1993). The evaluation of synergies and antagonisms among pollutants, including their dose–response relationships, is a necessary foundation for progressing toward a multipollutant air quality management framework, as noted by the NRC Committee on Air Quality Management in the United States (NRC 2004b).

In summary, our selective review confirmed that synergisms (greater than additive effects) among air pollutants in causing measurable biological effects have been demonstrated in laboratory studies of humans and animals. The limited sample of studies encompassed by this review served to answer this question, and it might be expected that a more comprehensive review of the air pollution literature would reveal additional evidence. Our review also identified evidence for additive and less than additive responses to combined exposures to multiple pollutants, sometimes among different outcome measures in studies in which certain outcomes revealed synergisms, and sometimes among the same outcomes at different times after exposure. To our knowledge, there has been no systematic review of all literature on the health effects of air pollution for evidence of pollutant interactions; thus, we have limited insight into the overall balance of the effects of the many possible combinations of pollutants. Although little, if any, evidence of synergisms has been developed at common environmental exposure levels, the possibility of both synergisms and antagonisms needs to be considered. In the absence of relevant evidence to the contrary, the assumption of additive effects appropriately remains the default for regulatory risk assessment purposes. However, considering the likelihood that both synergisms and antagonisms result from environmental exposures and their importance to accurate assessments of risk, evaluations of pollutant interactions by both epidemiologic and laboratory research approaches will be critical for developing a stronger foundation for multipollutant air quality management.

## Figures and Tables

**Table 1:** Studies demonstrating synergy between O_3_ and other pollutants.

Subject	O_3_	Other exposure	Synergistic effect	Reference
Young adult women	480 ppb × 2 hr	270 ppb peroxyacetyl nitrate × 2 hr	Forced expiratory variables	Horvath et al. 1986
Older men and women	450 ppb × 2 hr	600 ppb NO_2_ × 2 hr	Cardiac output and stroke volume	Drechsler-Parks 1995
Rat	600 ppb × 4 hr × 2 days	500 μg/m^3^ 0.3 μm H_2_SO_4_ × 4 hr × 2 days 500 μg/m^3^ 0.06 μm H_2_SO_4_ × 4 hr × 2 days	Alveolar epithelial proliferation Volume density of injured alveoli	Kimmel et al. 1997
Rat	150 ppb × 4 hr × 3 days × 40 weeks	50 μg/m^3^ HNO_3_ × 4 hr × 3 days × 40 wk	Lung polyamines	Sindhu et al. 1998
Rat	800 ppb × 4 hr	5,000 or 48,000 μg/m^3^ resuspended urban PM	Lung cell proliferation	Vincent et al. 1997
Rat	800 ppb × 4 hr	57,000 μg/m^3^ resuspended urban PM	Lung cell proliferation	Adamson et al. 1999
Guinea pig	1,500 ppb × 1 hr	2 puffs of 33% cigarette smoke	Dynamic lung compliance and resistance	Wu et al. 1997
Mouse	500 ppb × 24 hr	30,000 μg/m^3^ sidestream cigarette smoke × 6 hr × 3 days	Bronchoalveolar lavage cells and TNFα	Yu et al. 2002
Mouse	1,000 ppb × 24 hr	37.5 EU endotoxin × 10 min	Bronchoalveolar lavage IL-1 and IL-6	Johnston et al. 2002
Rat	500 ppb × 8 hr × 3 days	100 μg intranasal endotoxin × 2 days	Nasal epithelial mucosubstance	Fanucchi et al. 1998
Rat	500 ppb × 8 hr × 3 days	100 μg intranasal endotoxin × 2 days	Nasal epithelial mucosubstance	Wagner et al. 2001a
Rat	500 ppb × 8 hr × 3 days	100 μg intranasal endotoxin × 2 days	Nasal epithelial mucosubstance	Wagner et al. 2001b
Rat	500 ppb × 8 hr × 2 days	2 or 20 μg intranasal endotoxin × 2 days	Bronchoalveolar lavage neutrophils	Wagner et al. 2003
Rat	500 ppb × 8 hr × 3 days	50 μL intranasal 1% ovalbumin × 3 days	Nasal epithelial mucosubstance	Wagner et al. 2002

**Appendix I:** Potential interactions among pollutants.

Additivity: effect of the combination equals the sum of individual effects.
Synergism: effect of the combination is greater than the sum of individual effects.
Antagonism: effect of the combination is less than the sum of individual effects.
Inhibition: a component having no effect reduces the effect of another component.
Potentiation: a component having no effect increases the effect of another component.
Masking: two components have opposite, cancelling effects such that no effect is observed from the combination.

“Effect” means the observed expression of the particular health outcome in question. A combination of pollutants could have different interactions for different outcomes. The interaction could occur at any level of biological pathway from exposure to expression of the outcome (U.S. EPA 2000).
